# Gut microbiome and serum metabolome alterations in osteosarcoma patients

**DOI:** 10.3389/fmicb.2025.1616603

**Published:** 2025-07-17

**Authors:** Chao Li, Yu Chen, Weitao Yao, Peng Zhang, Xin Wang, Guoxin Qu, Zhigang Ren, Jiaqiang Wang

**Affiliations:** ^1^Department of Orthopaedic Surgery, The Affiliated Cancer Hospital of Zhengzhou University, Zhengzhou, China; ^2^School of Medicine, Sias University, Zhengzhou, China; ^3^Precision Medicine Center, The First Affiliated Hospital of Zhengzhou University, Zhengzhou, China

**Keywords:** osteosarcoma, gut microbiota, serum metabolites, characteristics, diagnostic biomarker

## Abstract

**Background:**

Gut microbiota has been shown to initiate tumorigenesis and cancer metastasis in multiple cancer types. However, the functional alterations of gut microbiota and their association with metabolism in osteosarcoma patients remain largely unexplored. This study aimed to characterize the gut microbiota and serum metabolite profiles in osteosarcoma patients, evaluate the diagnostic potential of gut microbiota and serum metabolites for osteosarcoma, and explore their correlations.

**Methods:**

We collected 128 fecal and 181 serum samples from osteosarcoma patients, paired with matched healthy controls. 16S rRNA sequencing and untargeted metabolomics were applied to analyze gut microbiota and serum metabolism with significantly altered abundance in patients with osteosarcoma. Models based on gut microbiome or serum metabolites were established and evaluated in an independent validation cohort.

**Results:**

The gut microbial diversity decreased in osteosarcoma patients compared to healthy individuals. Principal component analysis identified 33 microbial species that exhibited significant changes in osteosarcoma patients. Of note, the relative abundance of *Alloprevotella* and *Prevotella* increased in these patients. This distinct alteration in gut microbiota was accompanied by functional changes in pathways related to glycan degradation, pentose and glucuronate interconversions, the citrate cycle, and fructose and mannose metabolism during osteosarcoma progression. Furthermore, metabolomic analyses revealed a distinct distribution of serum metabolites in osteosarcoma patients compared to healthy controls. These metabolites were correlated with cancer’s carbon metabolism, glucagon signaling, and the citrate cycle pathways. Combined with the enrichment analysis results, gut microbiota and serum metabolites were associated with carbohydrate-related metabolism in osteosarcoma patients. Importantly, classifiers utilizing 3 optimal microbial markers (6 serum metabolites) demonstrated strong diagnostic efficiency in distinguishing osteosarcoma patients from healthy controls across various cohorts.

**Conclusion:**

This study thoroughly analyzed gut microbiota and serum metabolites in osteosarcoma patients, exploring their correlations and facilitating the establishment of a diagnostic model.

## Introduction

Osteosarcoma is the most common primary malignant bone tumor in children and adolescents, accounting for 67% of all primary malignant tumors of bone (Mirabello et al., 2009; Beird et al., 2022). Osteosarcoma is a high malignancy among all bone cancers, exhibiting aggressive growth and early metastasis to distant sites (Kager et al., 2003; Gorlick et al., 2013). For patients presenting with localized disease at diagnosis, standard neoadjuvant chemotherapy combined with surgical resection yields a 5-year survival rate of 70%. Metastatic disease, either at diagnosis or at the time of recurrence, portends a poor prognosis with a survival rate of 20% (Gill and Gorlick, 2021). Moreover, patients with osteosarcoma usually have non-specific clinical symptoms, which delay diagnosis until the tumors have reached an advanced stage and often result in a poor prognosis. Early detection and differentiation of osteosarcoma remain significant clinical challenges. Notably, the underlying pathophysiology of osteosarcoma remains largely unclear. Therefore, exploring novel diagnostic markers and effective therapies is crucial for improving patients’ prognoses.

The gut microbiota, which comprises most human microbial communities, is now considered a critical element in regulating host health and disease. Because of the advancement of molecular techniques, including metagenomic, metabolomic, lipidomic, and meta transcriptomic approaches, the intricate interactions between the human host and gut microorganisms are progressively being deciphered and characterized. Nowadays, mounting studies have highlighted the crucial role of gut microbiota in various diseases, including chronic kidney disease (Ren et al., 2020; Wang et al., 2023), type 2 diabetes (T2D) (Qin et al., 2012), colorectal cancer (CRC) (Chen et al., 2022; Sun et al., 2024), and pancreatic cancer (Mendez et al., 2020; Li et al., 2021), indicating its involvement in regulating pathways linked to immunity, energy metabolism, lipid metabolism, and glucose regulation (Eckburg et al., 2005; Rath and Dorrestein, 2012; Kundu et al., 2017; Levy et al., 2017; Dominguez-Bello et al., 2019). T2D has been associated with the outgrowth of facultative opportunistic pathogens (Qin et al., 2012), particularly oxidative stress resistance and perturbations in metabolites such as short-chain fatty acids and insulin resistance (Cani et al., 2007; Cani et al., 2009). CRC, the most prevalent gastrointestinal malignancy, has been strongly linked to disturbed gut microbiota with specific bacteria, such as *Fusobacterium nucleatum*, *Bacteroides fragilis*, and *Escherichia coli* (Chen et al., 2022; Sun et al., 2024). These microbial variations were involved in the aberrantly elevated concentrations of the secondary bile acid and deoxycholic acid, thereby suppressing CD8^+^ T cell responses to maintain tumorigenesis in CRC (Cong et al., 2024). Our previous research has verified the altered gut microbiota in COVID-19 (Ren et al., 2021), pancreatic carcinoma (Ren et al., 2017), hepatocellular carcinoma (Ren et al., 2019; Rao et al., 2020), and cholangiocarcinoma (Rao et al., 2021).

Metabolic changes can be indicators of osteosarcoma progression. The gut microbiota is crucial in regulating multiple aspects of metabolic disorders [26]. Microbial metabolites interact with host cells to activate or inhibit signaling pathways, impacting host health. This regulatory mechanism relies on the production of diverse metabolites and their interactions with host cell receptors, resulting in potential beneficial or detrimental effects on the host’s overall health. Recent animal studies have revealed correlations between osteosarcoma and gut microbiota. For instance, *Li et al*. demonstrated that *Alloprevotella* and *Rikenellaceae* were upregulated, while *Muribaculum*, *Klebsiella*, *Colidextribacter*, *and Lachnospiraceae* were downregulated in a BALB/c nude mouse model of osteosarcoma. Furthermore, *Alloprevotella*, *Rikenellaceae*, and *Muribaculum* abundances correlate with amino acid metabolism, particularly histidine metabolism (Li et al., 2024). However, none of the work has parallelly investigated gut microbiotas and serum metabolites in patients with osteosarcoma, as most previous studies have focused exclusively on either serum or fecal metabolites (Zhang et al., 2010; Lv et al., 2020).

Therefore, we used 16S rRNA MiSeq sequencing and serum metabolome analysis to explore the alterations in gut microbiota and their cross-talk with osteosarcoma-associated metabolism throughout the progression of osteosarcoma.

## Materials and methods

### Study participants and data collection

**FIGURE 1:**
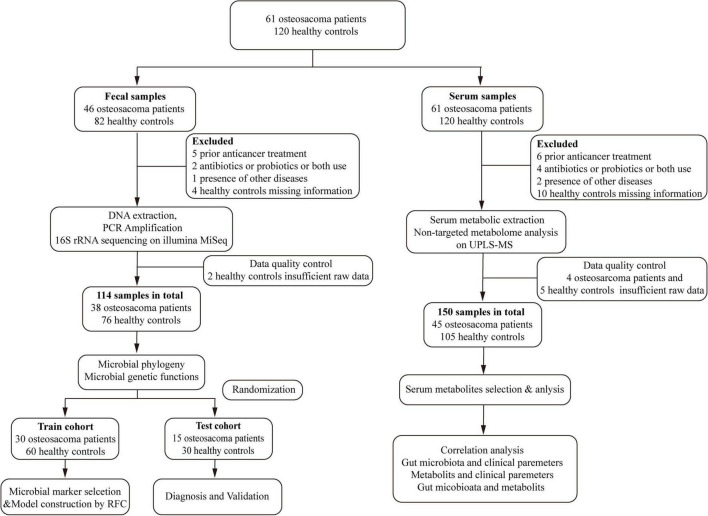
Study design and flow diagram. A total of 309 samples were collected. After applying the inclusion and exclusion criteria, 264 samples were ultimately selected for further analysis, including 114 fecal samples (38 OSs and 76 HCs) and 150 serum samples (45 OSs and 105 HCs). We initially examined the gut microbiota and serum metabolomics in OSs and HCs to identify significant microbial or serum metabolic markers. Subsequently, the fecal or serum samples were randomly assigned to either a training cohort or a test set. In the training cohort, prediction classifiers were developed using a fivefold cross-validation random forest model, which was then validated for diagnostic effectiveness in the test cohort. Additionally, we analyzed correlations between the gut microbiome and serum metabolomics, as well as the relationships between the gut microbiome or serum metabolites and clinical parameters. OSs, osteosarcoma patients; HCs, healthy controls; RFC, random forest classifier.

This study was conducted following the principles of the Declaration of Helsinki (Ren et al., 2019). From December 2020 to December 2022, 309 samples were collected, including 128 fecal and 181 serum samples (Figure 1). The trial procedures and eligibility criteria were reported previously (Ren et al., 2019). This study was approved by the Institutional Ethics Committee of the Affiliated Cancer Hospital of Zhengzhou University (Approval number: 2021-KY-0148-001). All participants provided written informed consent before participating in the study.

After applying all selection criteria, 264 samples were selected for the study. The fecal samples underwent thorough 16S rRNA MiSeq sequencing, while the serum samples were analyzed using ultra-performance liquid chromatography-mass spectrometry (UPLC-MS). In addition, key baseline factors for the participants included in this analysis encompassed age, sex, body mass index (BMI), primary tumor location and size, Enneking stage, and metastasis (Table 1).

**TABLE 1:** Demographic and clinical characteristics of study participants.

Clinical indices	Fecal sample		*p*-value	Serum sample		*P*-value
	Healthy controls (*n* = 76)	osteosarcoma (*n* = 38)		Healthy controls (*n* = 105)	Osteosarcoma (*n* = 45)	
Age (years)	16.18 ± 5.44	16.75 ± 7.23	0.671	15.87 ± 4.83	16.40 ± 5.47	0.552
Sex (female/male)	18/20	27/49	0.22	54/51	20/25	0.479
BMI	19.78 ± 4.12	20.94 ± 4.15	0.159	20.11 ± 3.89	20.05 ± 4.32	0.932
Tumor site (lower/upper extremity)		35/3			39/6	
Tumor volume (cm^3^)	−	292.64 ± 304.64		−	303.71 ± 319.24	−
Enneking stage						
Stages IIA-IIB	−	32(84.21%)		−	37(82.22%)	−
Stages IIIA-IIIB	−	6(15.79%)		−	8(17.78%)	−
WBC (10^∧^9/L)	5.58 ± 1.17	5.23 ± 1.35	0.158	5.82 ± 1.55	5.31 ± 1.40	0.062
RBC (10^∧^12/L)	4.57 ± 0.43	4.64 ± 0.45	0.449	4.63 ± 0.44	4.66 ± 0.48	0.727
Hemoglobin (g/L)	135.61 ± 16.29	140.08 ± 13.64	0.148	137.20 ± 16.04	140.93 ± 14.51	0.181
Platelet (10^∧^9/L)	249.43 ± 52.51	235.84 ± 42.72	0.17	260.67 ± 52.68	237.13 ± 41.54	0.009
Albumin (g/L)	48.34 ± 2.48	49.00 ± 2.63	0.194	48.69 ± 2.00	49.098 ± 2.63	0.304
Alkaline phosphatase (μ/L)	60.68 ± 13.77	257.77 ± 260.74	< 0.001	60.60 ± 14.09	262.27 ± 248.88	< 0.001
Scr (μmol/L)	70.94 ± 11.39	77.43 ± 10.79	0.004	72.59 ± 14.16	76.76 ± 10.51	0.078

BMI, body mass index; WBC, white blood cells; RBC, red blood cells; Scr, serum creatinine.

### Fecal sample collection and DNA extraction

Each participant was required to offer a fresh fecal sample between 6:00 and 8:00 a.m. Once received, each collected sample was divided into five equal parts of 200 mg and immediately stored at −80°C. Microbial genomic DNA was isolated from homogenized fecal suspensions (200 mg wet weight) using the E.Z.N.A.^®^ Stool DNA Kit (Omega Bio-Tek, Inc., GA) with the following critical modifications: Three cycles of bead-beating (0.1 mm zirconia/silica beads, 6.5 m/s for 45 s) using a FastPrep-24™ homogenizer (MP Biomedicals); extended incubation with InhibitorEX^®^ Buffer at 70°C for 10 min; post-extraction assessment via 1% agarose gel electrophoresis and Qubit™ dsDNA HS Assay (Thermo Fisher Q32851); DNA integrity was confirmed through PCR amplification of the 16S rRNA V4 region (positive control) and the lack of amplification observed in no-template controls. Qualified extracts were stored at –80°C in Tris-EDTA buffer (pH 8.0) with RNase A (10 μg/mL).

### PCR amplification, MiSeq sequencing, and data processing

PCR amplification targeting the hypervariable V3-V4 regions of bacterial 16S rRNA genes was performed using primers 341F (5′-CCTACGGGNGGCWGCAG-3′) and 805R (5′-GACTACHVGGGTATCTAATCC-3′), which were designed against *Escherichia coli* reference positions 341–805. Amplicons were size-selected (550–650 bp) using Hieff NGS DNA Selection Beads (YeasenBiotech Co., Ltd., China), followed by dual-index library construction with the TruSeq DNA PCR-Free Kit (Illumina). Paired-end sequencing (2 × 300 bp) was conducted on the Illumina MiSeq platform by Mobio Biomedical Technology (Shanghai). Raw sequencing data were processed by FLASH software (version 1.2.10) (Magoč and Salzberg, 2011). All sequencing data from this study are available in the Sequence Read Archive under BioProject PRJNA1184406.

### Operational taxonomic unit clustering and taxonomic annotation

Amplicon sequence variants (ASVs) were clustered into operational taxonomic units (OTUs) using the UPARSE pipeline (version 11)^1^ with a 97% sequence identity threshold (Edgar, 2013). OTUs were annotated at various taxonomic levels, including phylum, class, order, family, and genus, using the RDP Classifier V. 2.626^2^ against the SILVA^2^16S rRNA database (Wang et al., 2007).

### Bacterial diversity and taxonomic analysis

Shannon and Simpson indices and observed OTUs via mother 7 (v.1.42.1) were used to assess bacterial α diversity. The R package was utilized for bacterial β diversity analysis, and results were visualized through PCA and PCoA. Heatmaps were created using the R heatmap package.

PERMANOVA was employed to compare the gut microbiota of osteosarcoma patients and healthy individuals, focusing on identifying distinct characteristics, differential abundance, and multivariable correlations. Taxonomic discrimination was further analyzed using the LEfSe method (Segata et al., 2011), which highlighted key bacterial species that differentiated the gut microbiota of osteosarcoma patients from that of healthy controls based on a normalized relative abundance matrix (Ling et al., 2014). Finally, the potential variations in metabolic pathways were investigated using PICRUSt, which involved comparing data from 16S rRNA gene sequencing with the KEGG database.

### Identification of the OTU biomarkers and construction of probability of disease

The Wilcoxon rank-sum test was utilized to pinpoint significant biomarkers in the gut microbiome for further investigation. Subsequently, an optimal selection of OTUs was made utilizing a random forest model with fivefold cross-validation, as detailed in previous research (Heitner et al., 2010). For performance analysis of the classification models, we used the probability of disease (POD) index and Receiver Operating Characteristics (ROC) with the area under the curve (AUC) (Robin et al., 2011).

### Metabolomics preparations for serum samples

Blood samples were carefully processed to isolate the serum by centrifuging at 3,000 rpm for 10 min. For each sample, 100 μL of serum was mixed with 400 μL of 100% methanol (stored at –20°C) from each well in a 96-well plate and homogenized. After centrifugation (12,000 × g, 15 min, 4°C), the supernatants were dehydrated under N_2_ at 35°C. Dried extracts were reconstituted in 150 μL of 4 ppm 2-chlorophenylalanine solution (stored at –20°C), vortexed for 5 min, and filtered using a 0.22-μm PVDF filter for LC-MS detection. A pooled quality control sample was created from a small amount of each sample to ensure analytical consistency.

### UPLC-MS-based metabolomics data acquisition and analysis

The analysis employed acetonitrile (B3) and 5 mM ammonium formate (A3) as the phases. Metabolites were detected using an Orbitrap Exploris 120 from Thermo Fisher Scientific, equipped with an ESI ion source [34]. Subsequently, LC/MS raw data were converted to mzXML format through MSConvert [35] and processed using R XCMS for feature detection [36], retention time correction, and alignment. Identification of serum metabolites was performed by comparing against databases such as HMDB [37], Massbank [38], and KEGG [39]. Data were analyzed using the R package “ropls” (v1.22.0). To decipher metabolic profile variances between the groups, Partial Least Squares Discriminant Analysis (PLS-DA) and Orthogonal Partial Least Squares Discriminant Analysis (OPLS-DA) were utilized. Significant metabolites were defined based on a variable importance in projection (VIP) value > 1 and a false discovery rate < 0.05. The Wilcoxon rank-sum test was applied to compare metabolite levels between the groups.

### Statistical analysis

Continuous variables in the two groups were compared using either the Student’s *t*-test or the Wilcoxon rank-sum test, while categorical variables were evaluated with the χ^2^ test or Fisher’s exact test. The Spearman rank correlation test was used to determine statistical correlations. The level of statistical significance for two-tailed *p*-values was set at *p* < 0.05. Statistical analyses were carried out in SPSS v. 22.

## Results

### Study design and characteristics of the participants

Following the thorough application of strict criteria, 264 samples were selected for analysis, including 114 fecal samples (38 OSs and 76 HCs) and 150 serum samples (45 OSs and 105 HCs). Initially, we characterized the gut microbiota and serum metabolomics in patients with osteosarcoma and HCs to identify key microbial and serum metabolic markers. Subsequently, fecal and serum samples were randomly allocated into the training and test cohort. In the training cohort, predictive classifiers were constructed using a fivefold cross-validation random forest model, which was subsequently assessed for diagnostic performance in the test cohort (Figure 1). Additionally, correlations among the gut microbiome, serum metabolites, and clinical parameters were analyzed.

This study summarized and compared the demographic features of OSs and HCs (Table 1). Most OS cases (approximately 90%) were found in the lower extremities, while a smaller proportion (10%) were in the upper extremities. 80% of osteosarcoma patients were classified as stage II according to the Enneking Staging System. Interestingly, levels of alkaline phosphatase levels were markedly elevated in OSs when compared to HCs (*p* < 0.001). However, there were no notable differences in body mass index (BMI) or levels of various blood biochemical parameters like white and red blood cells, etc., as illustrated in Table 1.

### Gut microbial alterations in patients with osteosarcoma

**FIGURE 2:**
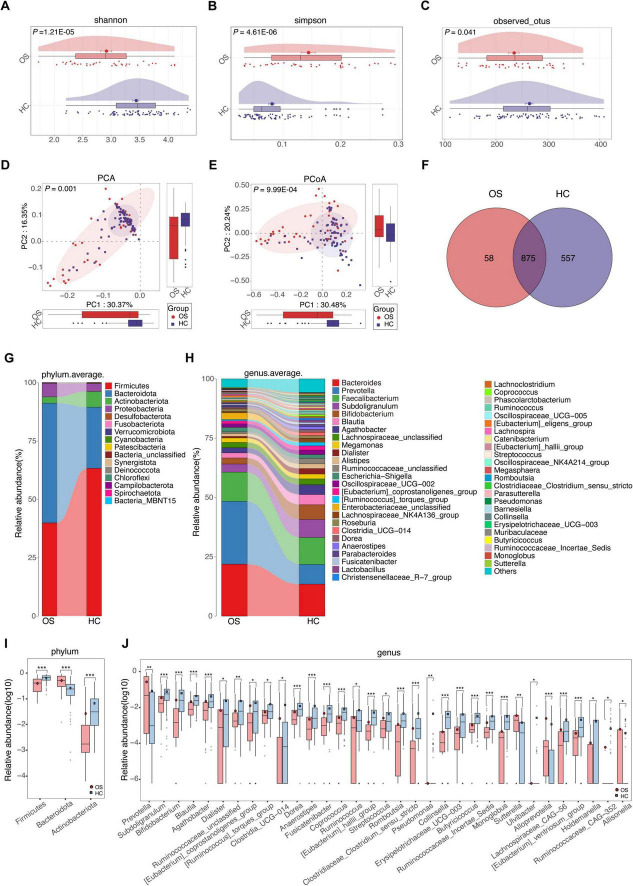
Gut microbial alterations in patients with osteosarcoma. Gut microbial alpha diversity was estimated using the Shannon index **(A)**, the Simpson index **(B)**, and the observed OTUs **(C)** in OSs (*n* = 38) and HCs (*n* = 76). The distribution of gut microbiota in the two groups was depicted by PCA **(D)** and PCoA analysis **(E). (F)** A Venn diagram illustrated that 875 out of 1,490 gut bacterial OTUs were shared between the two groups, while 58 OTUs were unique to the Oss. **(G)** The abundances of bacterial taxa in both groups are shown at the phylum level. **(H)** The abundance of bacterial taxa in both groups was displayed at the genus level. **(I)** Significant differences emerged in the abundances of discriminatory phyla between OS (red) and HC (blue). **(J)** Significant differences emerged in the abundances of discriminatory genera between OS (red) and HC (blue). OUT, operational taxonomic unit; PCA, principal component analysis; PCoA, principal coordinate analysis; *p < 0.05; **p < 0.01; ***p < 0.001.

To investigate the gut microbial characteristics in OSs, 16S rRNA MiSeq sequencing was performed on fecal samples from both OSs and HCs. The alpha diversity of gut microbes, as measured by the Shannon index, Simpson index, and observed OTUs, was significantly decreased in OSs compared to HCs (all *p* < 0.05) (Figures 2A–C and Supplementary Figure S2). Significant differences in composition were observed between the gut microbial communities of OSs and HCs in the PCA and PCoA analyses (all *p* < 0.001). The gut microbiota of HCs clustered together, while that of OSs was more heterogeneous, partially overlapping with that of the healthy individuals (Figures 2D,E). Venn diagram showed that 875 of the total 1,490 bacterial OTUs were shared between the two groups, while 58 unique OTUs were identified in OSs (Figure 2F). Furthermore, gut microbial composition and variation were analyzed, identifying 35 critical OTUs as distinguishing lineages between OSs and HCs (Supplementary Figures S1; Supplementary Data S2). Among these, 10 OTUs were elevated in OSs, while 25 were reduced compared to HCs.

Significant differences in gut microbial profiles between the OSs and HCs groups were analyzed at both the phylum and genus levels. At the phylum level, *Firmicutes*, *Bacteroidota*, *Actinobacteria*, and *Proteobacteria* predominated in both groups, accounting for about 90% of the microbiota (Figure 2G). In comparison to the HCs group, the OSs group exhibited a significant increase in *Bacteroidota*, accompanied by a decrease in *Firmicutes* and *Actinobacteria* (Figure 2I; Supplementary Data S3). At the genus level, *Prevotella*, *Sutterella*, *Alloprevotella*, and *Allisonella* were enriched, whereas 29 genera, including *Bifidobacterium*, *Butyricicoccus*, *Coprococcus*, and *Lachnospiraceae_CAG-56*, were remarkably reduced in the OSs group (Figures 2H,J; Supplementary Data S4). Furthermore, we compared the gut microbial composition between OSs and HCs at the class, order, and family levels. The abundance and composition of the bacterial community in each sample at the three levels are shown in Supplementary Figures S2–S4 and Supplementary Data S5–S7.

### Crucial gut bacteria and microbial functions related to osteosarcoma

**FIGURE 3:**
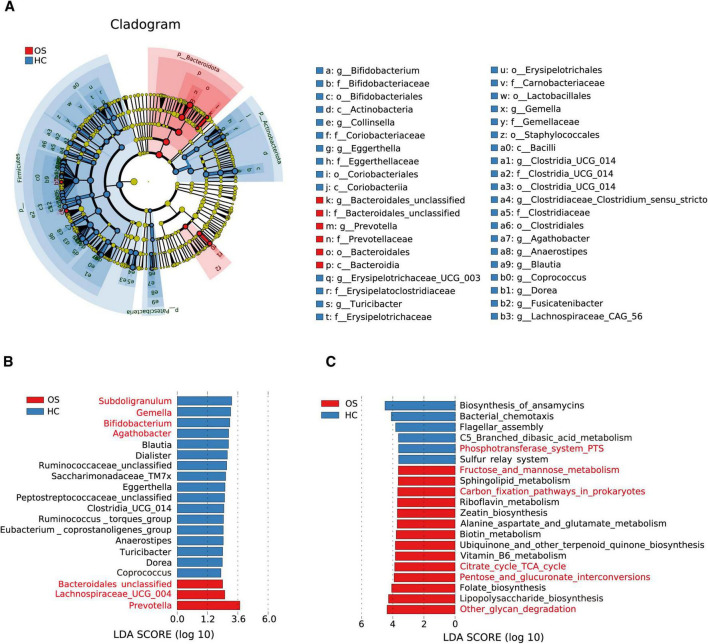
Crucial gut bacteria and microbial functions related to osteosarcoma. **(A)** A cladogram generated by the LEfSe method indicating the phylogenetic distribution of gut microbiota related to OSs (red) and HCs (blue). **(B)** Significant bacterial differences between the OSs and HCs groups based on LDA scores. **(C)** Prediction of key functional and metabolic pathways between the two groups.

To identify critical gut bacteria and microbial functions in osteosarcoma, we conducted Linear discriminant analysis Effect Size (LEfSe) to pinpoint specific bacterial taxa and predominant species associated with microbiota changes between OSs and HCs. The cladogram derived from the linear discriminant analysis (LDA) showed significant differences in gut microbial taxa between the groups (Figure 3A and Supplementary Data S8). At the genus level, OSs were characterized by the enrichment of *Prevotella*, *Lachnospiraceae UCG 004*, and *Bacteroidales unclassified* (LDA score (log10) > 3). Conversely, *Subdoligranulum*, *Gemella*, *Bifidobacterium*, and *Agathobacter* were dramatically depleted in the OSs group (LDA score (log10) > 3), as depicted in Figure 3B (Supplementary Data S9).

We utilized the Phylogenetic Investigation of Communities by Reconstruction of Unobserved States 2 (PICRUSt2) to predict the key functional and metabolic pathways of microbial communities based on their 16S rRNA gene content. 58 KEGG pathways differed markedly between the OSs and HCs groups (all *p* < 0.05, LDA score > 3, Supplementary Data S10). Among the 20 top predicted pathways (Figure 3C), six were related to carbohydrate metabolism, including glycan degradation, pentose, and glucuronate interconversions, the TCA cycle, carbon fixation in prokaryotes, and fructose and mannose metabolism, which were enriched in the OSs group. In contrast, the phosphotransferase system was more abundant in the HCs group.

### Non-invasive diagnostic model for osteosarcoma via the gut microbiota

**FIGURE 4:**
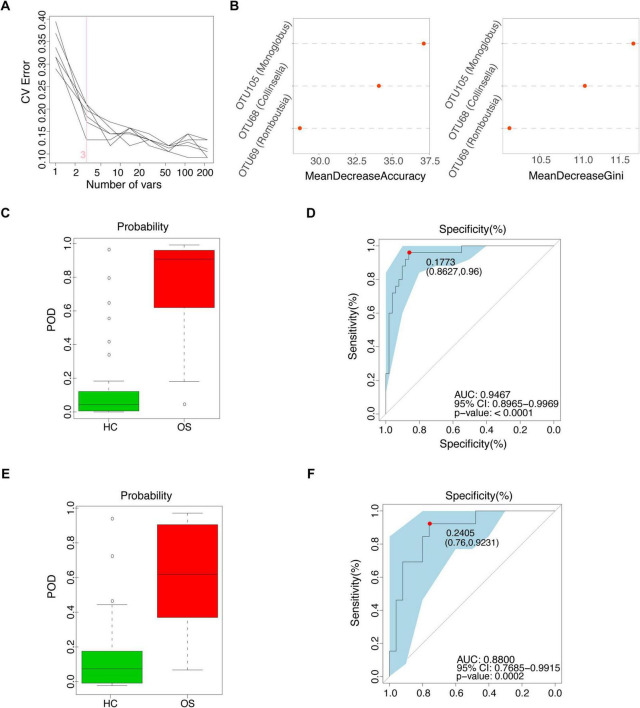
Non-invasive diagnostic model for osteosarcoma via the gut microbiota. **(A)** 3 OUT microbial markers identified as the optimal marker set by the random forest model. **(B)** Importance distribution map of the selected microbial markers in the model. **(C)** The POD value in the training cohort (25 OSs and 51 HCs). **(D)** ROC analysis of the selected microbial markers to discriminate OSs from HCs in the training cohort. **(E)** The POD value in the test cohort (13 OSs and 25 HCs). **(F)** ROC analysis of the selected microbial markers to discriminate OSs from HCs in the test cohort; POD probability of disease, AUC, area under the curve.

To investigate the diagnostic potential of gut microbiota composition concerning osteosarcoma, we developed a random forest classifier model based on gut OTUs from 25 OSs and 50 HCs in the training cohort, as shown in Figure 1. Three gut OTUs, including OTU68 (*Collinsella*), OTU69 (*Romboutsia*), and OTU105 (*Monoglobus*), were identified as the optimal marker set that achieved the lowest classifier error in the random forest cross-validation (Figures 4A,B). The relative abundance of these three OTUs in each sample was shown in Supplementary Data S11. The POD index was calculated using the identified optimal set of three OTUs for both training and validation cohorts. In the training phase, the POD value was significantly higher in the OSs group than in the HCs group, yielding an area under the curve (AUC) value of 94.67% (95% CI: 89.65–99.69%, *p* < 0.0001) (Figures 4C,D and Supplementary Data S12). In the validation phase, which consisted of 13 OSs and 26 HCs, the average POD index for OSs was markedly elevated compared to that for HCs, achieving an AUC value of 88.00% (95% CI: 76.85–99.15%, *p* = 0.0002), as demonstrated in Figures 4E,F (Supplementary Data S13). These findings suggest that the gut microbial classifier model could specifically distinguish osteosarcoma patients from the HCs group.

### Serum metabolomics alterations in patients with osteosarcoma

**FIGURE 5:**
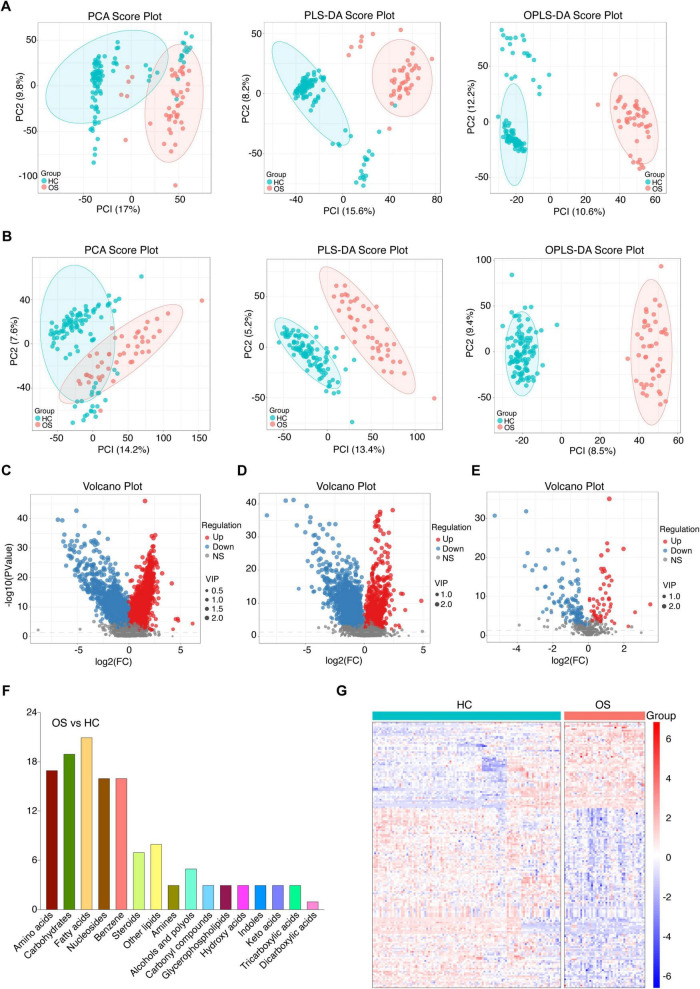
Serum metabolomics alterations in patients with osteosarcoma. **(A)** The positive ionic distribution of serum metabolites assessed by PCA, PLS-DA, and OPLS-DA in OSs (*n* = 61) and HCs (*n* = 120). **(B)** The negative ionic distribution of serum metabolites assessed by PCA, PLS-DA, and OPLS-DA in OSs and HCs. **(C)** Volcano plot illustrating changes based on the positive ionic distribution. **(D)** Volcano plot illustrating changes based on the negative ionic distribution. **(E)** Volcano plot depicting the distribution based on the secondary spectra of the fragment ions between the two groups. **(F)** The categories and number of differential serum metabolites identified from the OSs compared to HCs. **(G)** Heat map of the differential serum metabolites in the OSs and HCs groups. PLS-DA, partial least squares discrimination analysis; OPLS-DA, orthogonal partial least squares discriminant analysis.

Gut microbiota produces biologically active metabolites that can enter the bloodstream and regulate various physiological processes in humans (Dominguez-Bello et al., 2019). To further investigate the altered metabolomic profile in osteosarcoma, we conducted untargeted metabolomics via UPLC-MS. Serum samples were collected from 61 OSs and 120 HCs. The total ion chromatogram of the quality control samples showed a stable baseline in both groups (Supplementary Figure S5). As demonstrated in Figures 5A,B, PCA, PLS-DA, and OPLS-DA analyses in both positive and negative ion scanning modes revealed significant differences in serum metabolites between OSs and HCs.

To further characterize the altered serum metabolites in osteosarcoma progression, we performed a pairwise comparison using VIP values from OPLS-DA and *p-* values obtained from statistical analysis. Differential serum metabolites with consistent shift were shown in Figures 5C–E, with 57 metabolites enriched and 116 depleted in OSs compared to HCs. (Student’s *t*-test, *p* < 0.01, FC > 1.5, and VIP > 1, Figure 5E) As depicted in Figure 5F, fatty acids, carbohydrates, and amino acids were the predominant affected, accounting for 40% of the total altered metabolites. The OPLS-DA score plot presented a clear separation between OSs and HCs based on these serum metabolites (Figure 5G and Supplementary Data S14).

### Crucial metabolism-related signaling pathway analysis in osteosarcoma

**FIGURE 6:**
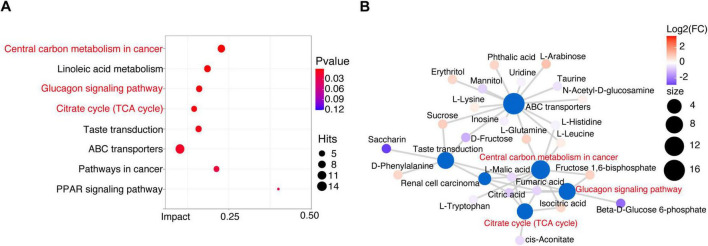
Crucial metabolism-related signaling pathway analysis in osteosarcoma. **(A)** Enrichment analysis of differential metabolic pathways. **(B)** Metabolic network analysis based on the enriched metabolic pathways and the KEGG database.

To explore the mechanism underlying the stage of osteosarcoma, we performed metabolic pathway enrichment analysis on differential metabolites between OSs and HCs. 40 metabolites were associated with 13 altered functional pathways, including central carbon metabolism in cancer, linoleic acid metabolism, and glucagon signaling pathway (Figure 6A and Supplementary Data S15). Of note, half of the top six pathways pertained to carbon metabolism in cancer, glucagon signaling pathway, and citrate cycle (TCA cycle) were closely linked to carbohydrate metabolism (Figure 6B). Interestingly, gut microbial carbohydrate metabolism, including pathways like other glycan degradation, pentose and glucuronate interconversions, citrate cycle (TCA cycle), and fructose and mannose metabolism, was significantly enriched in the OSs group (Figure 3C). These findings above imply that carbohydrate-related metabolic pathways play a crucial role in the progression of osteosarcoma.

### Identification of the serum metabolites classifier for osteosarcoma

**FIGURE 7:**
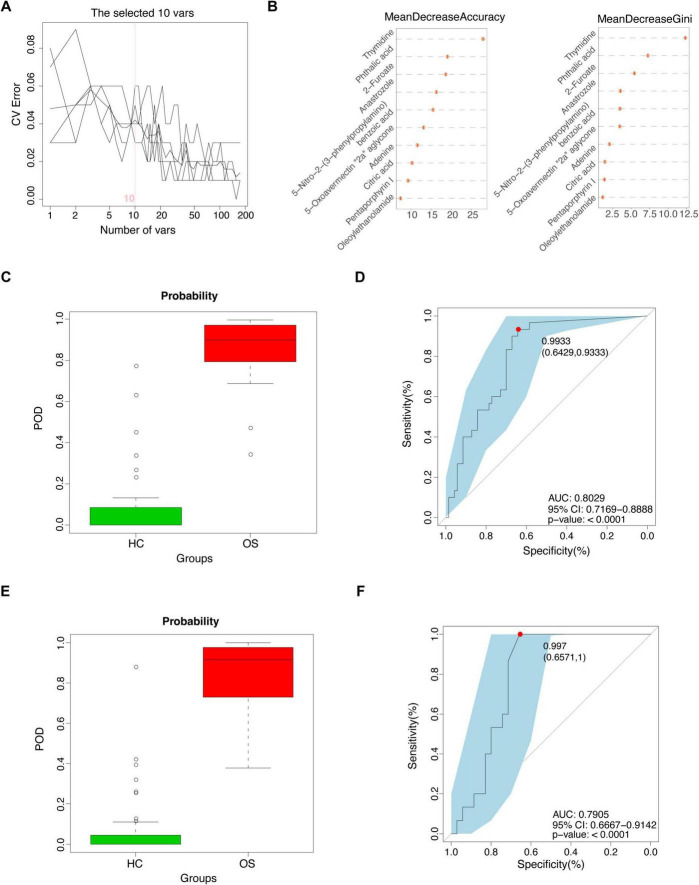
Identification of the serum metabolites classifier for osteosarcoma. **(A)** Ten serum metabolic markers identified as the optimal marker set by the random forest model. **(B)** Importance distribution map of the selected metabolic markers in the model. **(C)** The POD value in the training cohort (30 OSs and 70 HCs). **(D)** ROC analysis for the selected metabolic markers to distinguish OSs from HCs in the training cohort. **(E)** The POD value in the test cohort (15 OSs and 35 HCs). **(F)** ROC analysis for the selected metabolic markers to distinguish OSs from HCs in the test cohort; POD probability of disease, AUC, area under the curve.

Next, we investigated the potential of serum metabolites as diagnostic biomarkers for osteosarcoma by constructing a random forest classifier using differential serum metabolites from the training cohort (30 OSs versus 70 HCs). A fivefold cross-validation was employed to assess the robustness of the model. As a result, a panel of 10 serum metabolite biomarkers showed outstanding performance in discriminating OSs from HCs. Among these metabolites, four metabolites (phthalic acid, oleoylethanolamide, pentaporphyrin I, and thymidine) were enriched in OSs group, whereas 6 metabolites, including 5-nitro-2-(3-phenylpropylamino) benzoic acid, 5-oxoavermectin “2a” aglycone, 2-furoate, adenine, citric acid, and anastrozole, were depleted (Figures 7A,B). Notably, the POD index for osteosarcoma calculated from these 6 metabolites showed an increase significantly compared with that of the HCs, with an AUC of 0.8029 (95% CI 71.69–88.88%, *p* < 0.0001), as shown in Figures 7C,D (Supplementary Data S16).

Furthermore, validation with a test cohort (15 OSs and 35 HCs) confirmed the classifier’s effectiveness, yielding an AUC of 0.7905 (95% CI 66.67–91.42%) (Figures 7E,F and Supplementary Data S17). These data suggested that the serum metabolic classifier may differentiate OSs from HCs. Comparing the AUCs of both models revealed that the gut microbiota panel (AUC 0.8800–0.9467) outperformed the serum metabolite panel (AUC 0.7905–0.8029) in distinguishing osteosarcoma from HCs.

### Correlation among gut microbiome, serum metabolites, and clinical index in osteosarcoma

**FIGURE 8:**
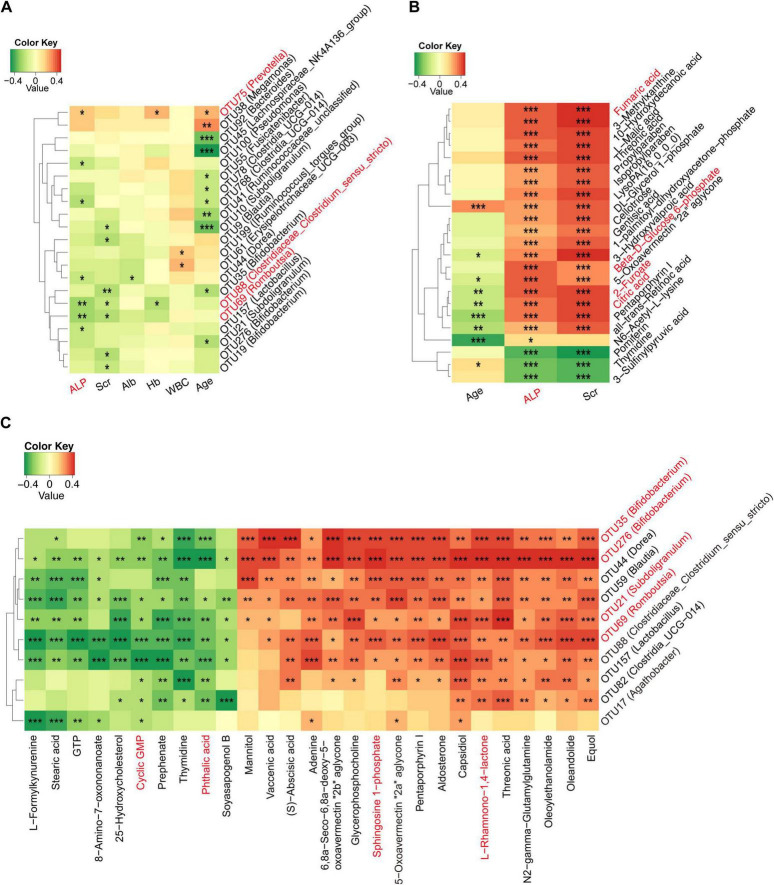
Correlation among gut microbiome, serum metabolites, and clinical indices in osteosarcoma. **(A)** Correlations between differing gut microbiota and clinical indicators in the progression of osteosarcoma. **(B)** Correlations between differing serum metabolites and clinical indicators in the progression of osteosarcoma. **(C)** Correlations between differing gut microbiota and serum metabolites in the progression of osteosarcoma. OTU, operational taxonomic unit; RBC, red blood cell count; Hb, hemoglobin; ALB, albumin; Scr, serum creatinine; ALP, alkaline phosphatase. *P < 0.05; **P < 0.01; ***P < 0.001.

We conducted a deeper analysis of the relationships among the gut microbiome, serum metabolites, and clinical indicators of osteosarcoma through Spearman correlation analysis. A total of 21 OTUs were identified as being linked to five clinical indicators (Figure 8A and Supplementary Data S18), including a positive correlation between ALP and osteosarcoma-increased gut microbiota (e.g., *Prevotella)* and a negative correlation with osteosarcoma-decreased gut microbiota, like *Romboutsia* and *Clostridiaceae*. Meanwhile, we examined the correlations between serum metabolites and clinical indicators in osteosarcoma and found three clinical indicators (ALP, Scr, and Age) closely related to 23 serum metabolites (Figure 8B and Supplementary Data S19). Intriguingly, ALP and Scr were positively associated with fumaric acid, beta-d-glucose 6-phosphate, 2-furoate, and citric acid, which were involved in carbohydrate metabolism.

Next, we investigated the relationship between varying gut microbes and metabolites in osteosarcoma. The results showed that osteosarcoma-decreased microbes were positively correlated with serum-depleted metabolites (Figure 8C and Supplementary Data S20). Conversely, negative correlations were observed between osteosarcoma-decreased microbes and serum-enriched metabolites. Specifically, osteosarcoma-decreased gut microbes (e.g., *Bifidobacterium*, *Subdoligranulum, and Romboutsia*) were positively associated with serum-enriched metabolites (e.g., L-Rhamnono-1,4-lactone and sphingosine 1-phosphate) and negatively correlated with serum-depleted metabolites (e.g., phthalic acid and cyclic GMP). All of the above findings indicated a potential interaction among the gut microbiome, serum metabolites, and clinical indicators in the progression of osteosarcoma.

## Discussion

Numerous studies have demonstrated that gut microbial dysbiosis is closely associated with various cancers, making it a novel target for diagnosis and therapy [21, 43, 44]. However, the role of gut microbiota and serum metabolites in patients with osteosarcoma requires further exploration. This study aimed to analyze the characteristics of gut microbiomes and metabolomics in OSs compared with HCs and identify crucial microbiota and metabolites. Then, we established predictive models based on the differential microbiota and metabolites, which reached a powerful diagnostic potential in distinguishing osteosarcoma from HCs.

Gut microbiota colonizing humans has been identified at the phylum level, including *Firmicutes*, *Bacteroidetes*, *Actinobacteria*, *Proteobacteria*, *Fusobacteria*, and *Verrucomicrobia*, among which *Firmicutes* and *Bacteroidetes* are the major phyla (Laterza et al., 2016). Consistent with these findings, we also found that *Firmicutes*, *Bacteroidetes*, *Actinobacteria*, and *Proteobacteria* were the predominant bacterial phyla in both OSs and HCs. Specifically, *Firmicutes* and *Actinobacteria* decreased significantly in OSs, whereas *Bacteroidetes* increased. In addition, analysis of the gut microbiota revealed that 33 microbial species significantly changed in OSs. The phylum *Bacteroidota*, particularly the genera *Alloprevotella* and *Prevotella*, had the highest abundance in the OSs group, followed by *Proteobacteria* (*Sutterella*) and *Firmicutes* (*Allisonella*). Emerging evidence suggests that some of these bacteria pose a substantial risk to human health. For instance, *Alloprevotella* and *Prevotella* have been implicated in tumorigenesis, and elevated levels have been shown to be associated with oral cavity cancer (Ganly et al., 2019; Liu X. et al., 2023), breast cancer (Liu E. et al., 2023), and pancreatic cancer (Chen et al., 2023). As opposed to the observed decrease of *Allisonella* in prostate cancer and bladder cancer (Mingdong et al., 2023), there was an increase in osteosarcoma. Such a unique alteration may constitute a characteristic gut microbiota signature distinguishing osteosarcoma from other cancers.

Subsequently, the KEGG enrichment analysis revealed that gut microbial dysbiosis in osteosarcoma was closely associated with several disordered pathways, including other glycan degradation, pentose and glucuronate interconversions, lipopolysaccharide biosynthesis, citrate cycle (TCA cycle), carbon fixation pathways in prokaryotes, and fructose and mannose metabolism pathways. These pathways involved inflammatory reactions, immune responses, and oxidative stress (Sepich-Poore et al., 2021; Hou et al., 2022). Thus, our work suggested that alterations in the gut microbiota associated with osteosarcoma may be attributed to the imbalanced function of inflammatory and immune responses. Research reveals a strong connection between the gut microbiome and several diseases, suggesting it may function as a non-invasive diagnostic tool for specific conditions. *Wang et al*. examined changes in the gut microbiota associated with chronic kidney disease (CKD) and developed a diagnostic model using gut microbial markers, achieving significant efficacy in differentiating CKD patients from healthy individuals (Wang et al., 2023). *Chen et al*. comprehensively described gut microbiota characteristics in pancreatic cancer and proposed potential microbial markers as non-invasive tools for its diagnosis (Chen et al., 2023). Our previous analyses have investigated that gut microbial markers possess strong diagnostic potential for hepatocellular carcinoma (Ren et al., 2019; Rao et al., 2020) and cholangiocarcinoma (Rao et al., 2021). Therefore, we established a diagnostic panel of three OTU biomarkers, which showed excellent performance in discriminating OSs from HCs in the training cohort with an AUC of 94.67 and 88.00% in the test cohort. These findings underscored gut microbiota dysbiosis as a promising diagnostic biomarker and a potential therapeutic strategy for osteosarcoma patients.

Dysregulated metabolism is a hallmark of cancer (Hanahan and Weinberg, 2011). The serum metabolome plays a vital role in mediating the impact of gut microbiota on host phenotypes in both health and disease conditions (Chen et al., 2021). Meanwhile, in this study, we observed a distinct distribution of serum metabolites in OSs compared to HCs, which was associated with carbon metabolism in cancer, the glucagon signaling pathway, and the citrate cycle (TCA cycle) pathways. Furthermore, the pathway and enrichment analyses of gut microbiota and serum metabolites revealed a connection to carbohydrate-related metabolism in OSs. This finding is consistent with recent reports emphasizing disrupted energy metabolism in Oss (Zhang et al., 2010; Dean et al., 2018; Lv et al., 2020). Then, we examined the diagnostic potential of serum metabolites and constructed a diagnostic panel consisting of six serum metabolite biomarkers (4 osteosarcoma and six depleted), which showed adequate accuracy for differentiating OSs from HCs (AUC = 0.8029). These results implicated the critical role of serum metabolites in osteosarcoma progression.

In addition, the correlation analysis revealed mutual interactions among gut microbiota, serum metabolites, and clinical indicators of osteosarcoma. Of note, the level of alkaline phosphatase was positively correlated with osteosarcoma-enriched *Prevotella* (*r* = 0.209, *p* = 0.025) as well as osteosarcoma-associated metabolites, including fumaric acid (*r* = 0.429, *p* = 0.000), beta-D-Glucose 6-phosphate (*r* = 0.403, *p* = 0.000), 2-furoate (*r* = 0.407, *p* = 0.000), and citric acid (*r* = 0.376, *p* = 0.000). These findings support previous studies that identified elevated alkaline phosphatase as linked to poorer overall survival in osteosarcoma patients (Ferrari et al., 2001; Bacci et al., 2006). Meanwhile, the osteosarcoma-decreased microbes were positively associated with serum-depleted metabolites, while negative correlations were observed between osteosarcoma-decreased microbes and serum-enriched metabolites. Collectively, these results deciphered a potential relationship among gut microbiota, clinical parameters, and metabolites that may contribute to the progression of osteosarcoma. However, further investigation is needed to determine the causal relationship and underlying mechanisms.

Despite the advantages outlined above, this study has several limitations. It was constrained by the absence of early stage cases, as most patients enrolled with osteosarcoma presented with advanced disease (stage II/III according to the Enneking staging system). This led to an insufficient sample size for stage-stratified analysis. Additionally, since this is a single-center study conducted in China, the effectiveness of the diagnostic models has not been validated across different regions.

## Conclusion

In summary, we comprehensively characterized the alterations in gut microbiota accompanied by distinct changes in the metabolomic profiling of osteosarcoma. Furthermore, the intricate interactions among osteosarcoma-related species, metabolites, and clinical indices were closely associated with osteosarcoma progression. Clinically, we established diagnostic models based on gut microbiota and serum metabolites that effectively differentiate osteosarcoma patients from healthy controls with high specificity. Therefore, our research highlights the critical role of gut microbes and metabolites in the development of osteosarcoma, providing new diagnostic and therapeutic targets for patients.

## Data Availability

All sequencing data is available in the NCBI Sequence Read Archive (SRA) under BioProject PRJNA1184406. Additionally, the raw numerical data that were used to create the figures in our manuscript are openly available in Supplementary Material 2: Supplementary Data S1–S20. The other data supporting the findings is available in the methods and from the corresponding author upon reasonable request.

## Footnotes

1
http://drive5.com/uparse/

2
http://rdp.cme.msu.edu/

## Supplementary material

Supplementary Data S1 Detailed values of the gut microbial diversity index and the observed OTUs in 76 HCs and 38 OSs.

Supplementary Data S2 The abundance and distribution of 35 OUTs in both groups.

Supplementary Data S3 Phylum-level difference between the two groups.

Supplementary Data S4 Genus-level differences between the two groups.

Supplementary Data S5 Class-level differences between the two groups.

Supplementary Data S6 Order-level differences between the two groups.

Supplementary Data S7 Family-level differences between the two groups.

Supplementary Data S8 Identified bacterial taxa and predominant bacteria in OSs (*n* = 38) and HCs (*n* = 76) through LEfSe analysis.

Supplementary Data S9 The LDA value and *p*-value associated with the genera significantly differed between the two groups.

Supplementary Data S10 The LDA value and *p*-value related to the microbial community gene function for samples from both groups.

Supplementary Data S11 Each optimal microbial marker’s output value in the training cohort.

Supplementary Data S12 The corresponding POD values for each sample in the training cohort.

Supplementary Data S13 The corresponding POD values for each sample in the test cohort.

Supplementary Data S14 Serum metabolites differ according to the VIP values from OPLS-DA and the *p*-values from statistical analysis.

Supplementary Data S15 Analysis of enriched differential metabolic pathways in both groups.

Supplementary Data S16 The POD value linked to each sample in the training cohort.

Supplementary Data S17 The POD values corresponding to each sample in the test cohort.

Supplementary Data S18 The *p*-value from Spearman’s correlation analysis related to variations in gut microbiota and clinical indicators in the progression of osteosarcoma.

Supplementary Data S19 The *p*-value from Spearman’s correlation analysis of differential serum metabolites in relation to clinical indicators during the progression of osteosarcoma.

Supplementary Data S20 Spearman’s correlation analysis revealed the *p*-value linking differential gut microbiota to serum metabolites in the progression of osteosarcoma.
