# Supplementary material for: Gut microbiome and serum metabolome alterations in osteosarcoma patients
Source: Front Microbiol. 2025 Jul 17;16:1616603. doi: 10.3389/fmicb.2025.1616603 (PMC12310664; doi:10.3389/fmicb.2025.1616603)
Supplement: Supplementary file 1 [file Supplementary_file_1.docx]

**Supplementary file 1**

**Figure S1.** The heat map illustrates the relative abundances of the discriminatory OTUs that differentiate OSs (n = 38) from HCs (n = 76). The columns for each sample display the relative abundance of the differential OTUs listed to the right of the figure. The heat map is plotted according to the relative abundance of each OTU, with blue indicating low abundance and red representing high abundance. Group information is shown above the plot: OSs are marked on the left with a red line, while HCs are represented on the right with a blue line. Each row corresponds to a single OTU. OSs, osteosarcoma patients; HCs, healthy controls; OUT, operational taxonomic unit.


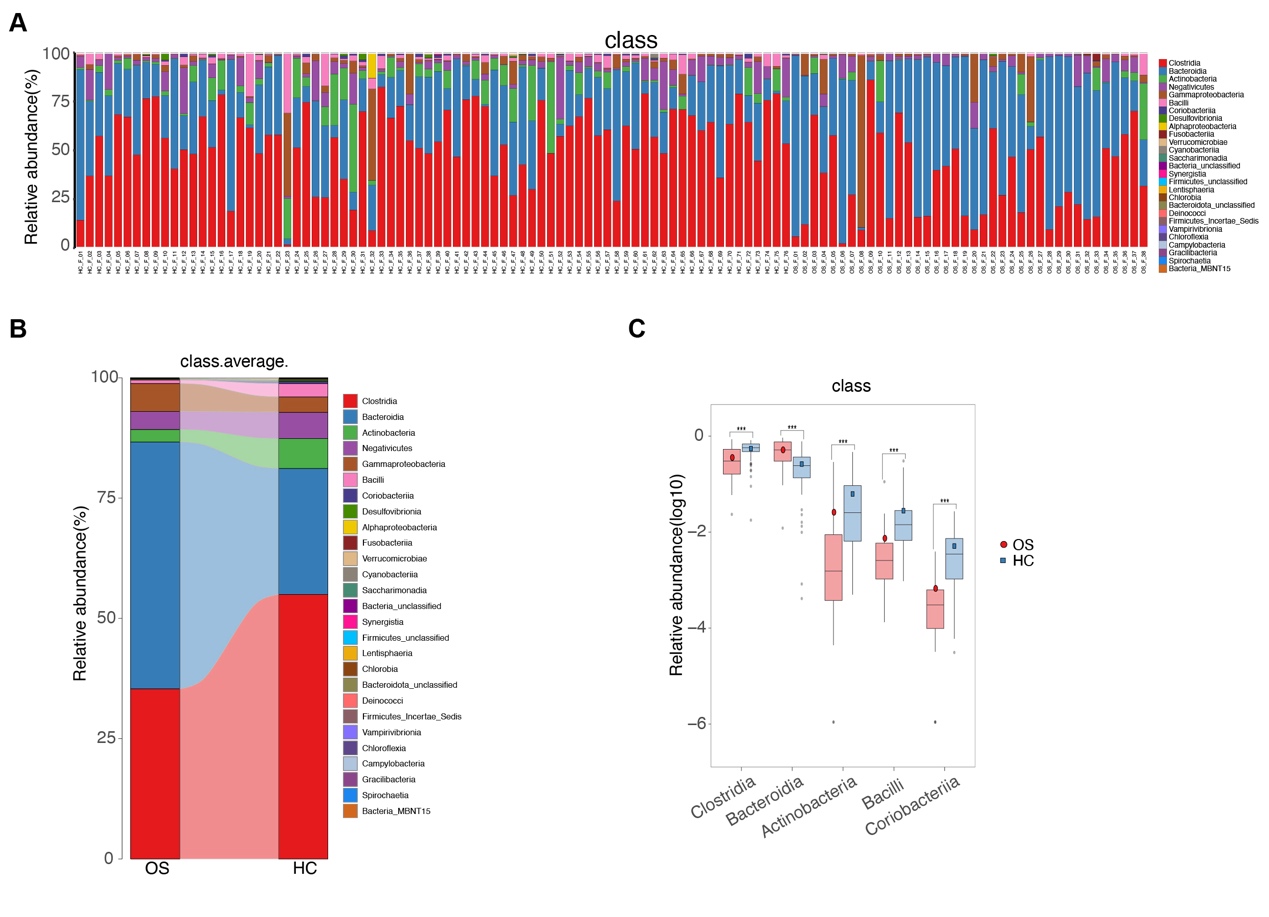


**Fig. S2. A.** Analyzed the bacterial community's composition and abundance in each sample at the class level, including 38 OSs and 76 HCs. **B.** Bacterial community composition and abundance at the class level for both groups. **C.** *Bacteroidia* showed significant enrichment at the class level, while three bacterial populations were notably reduced in OSs compared to HCs. ***, *p* < 0.001


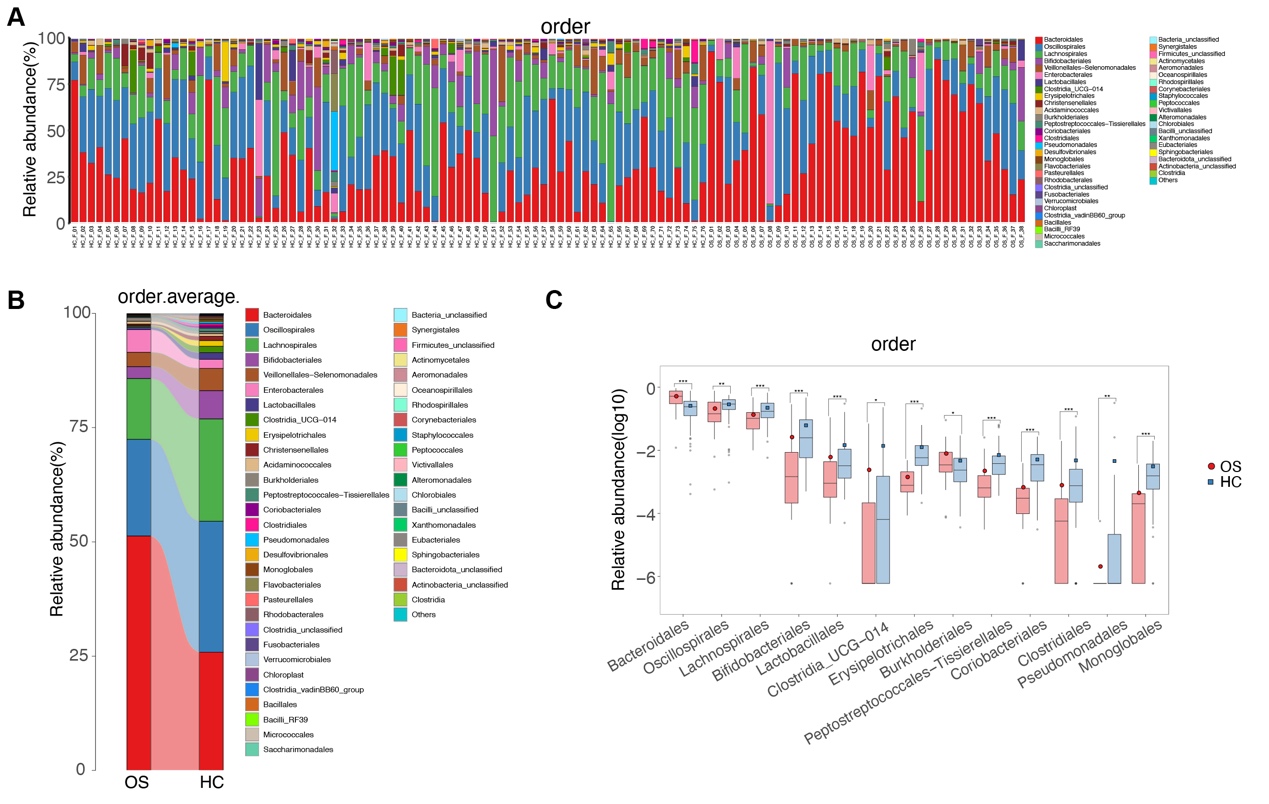


**Fig. S3. A.** Analyzed the bacterial community's composition and abundance in each sample at the order level, including 38 OSs and 76 HCs. B. Bacterial community composition and abundance at the order level in both groups. **C.** Two bacterial populations were enriched and eleven were reduced in OSs compared to HCs. *, *p* < 0.05, **, *p* < 0.01, ***, *p* < 0.001.


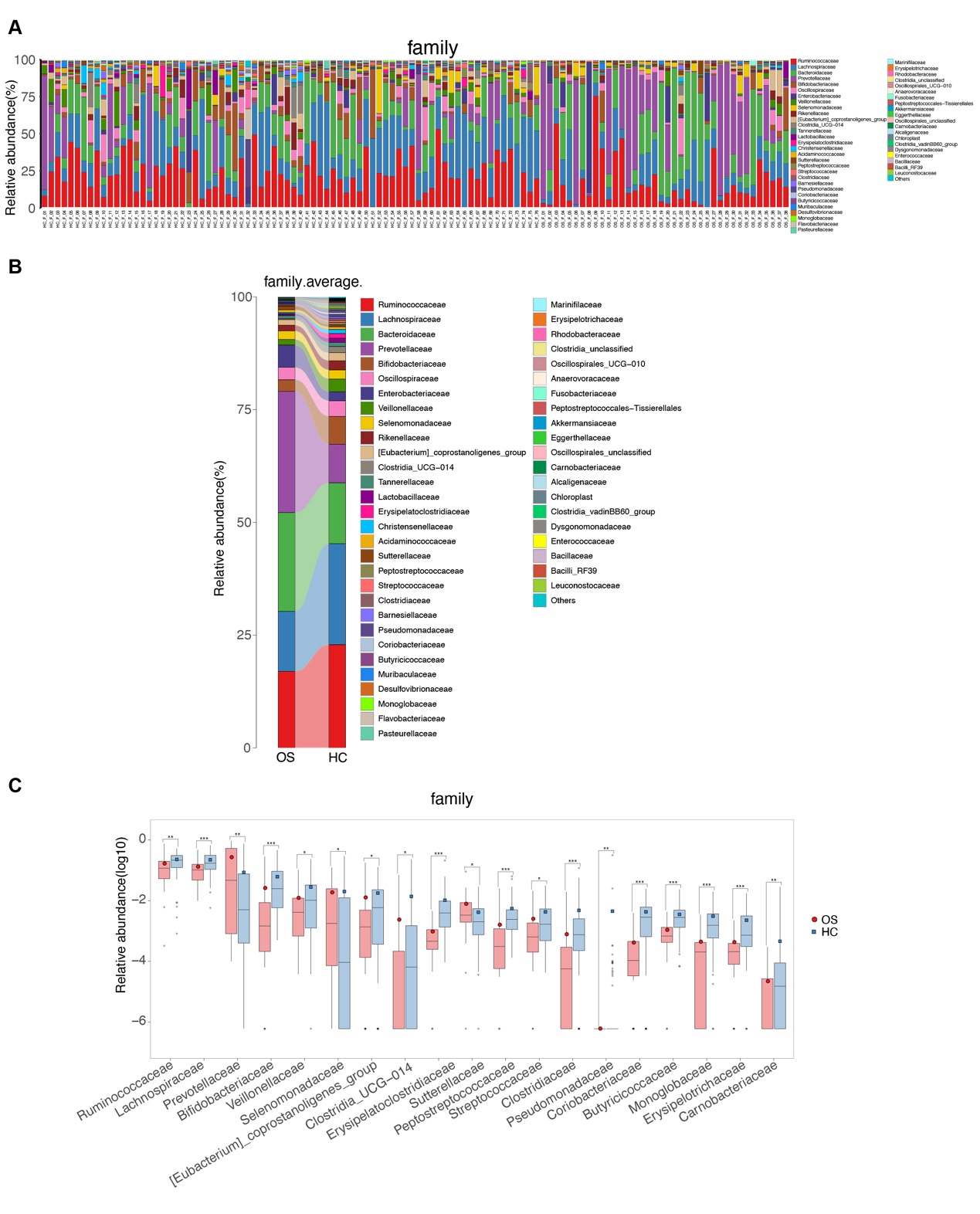


**Fig. S4. A.** Analyzed the bacterial community's composition and abundance in each sample at the family level, including 38 OSs and 76 HCs. B. Average composition and relative abundance at the family level in both groups. C. Three bacterial populations were significantly enriched, and sixteen were reduced in OSs compared to HCs. *, *p* < 0.05, **, *p* < 0.01, ***, *p* < 0.001.

**Figure S5.** Total ion chromatographic flow diagrams of QC samples in OSs and HCs.
